# Factors associated with viremia in people living with HIV on antiretroviral therapy in Guatemala

**DOI:** 10.1186/s12981-021-00400-9

**Published:** 2021-10-27

**Authors:** Dean W. Ortíz, Olivia Roberts-Sano, Hugo E. Marroquin, Lindsey Larson, Katherine B. Franco, Andrej Spec, Johanna R. Melendez, Rodolfo Pinzón, Ana J. Samayoa, Carlos Mejia-Chew, Jane A. O´Halloran

**Affiliations:** 1 Unidad de Atención Integral del VIH e Infecciones Crónicas del Hospital Roosevelt “Dr. Carlos Rodolfo Mejía Villatoro”, Calzada Roosevelt, 5ta. Calle, zona 11, Guatemala City, Guatemala; 2 Department of Medicine, Division of Infectious Diseases, Washington University School of Medicine, St. Louis, USA

**Keywords:** HIV, Treatment outcomes, Health risk behavior, Health services accessibility, Sustained virologic suppression, Gender

## Abstract

**Introduction:**

Viral suppression prevents HIV transmission and disease progression, but socio-economic and clinical factors can hinder the goal of suppression. We evaluated factors associated with viral non suppression (VNS) and persistent viremia (PV) in people living with HIV (PLHIV) receiving antiretroviral therapy (ART) in Guatemala.

**Methods:**

We conducted a cross sectional analysis using data from an ongoing cohort of PLHIV attending the largest HIV clinic in Guatemala. Univariable and multivariable analyses were conducted between PLHIV with viral suppression and detectable viremia. VNS was defined as most recent HIV RNA ≥ 200 copies/ml and PV as two consecutive HIV RNA ≥ 200 copies/ml.

**Results:**

Of 664 participants, 13.3% had VNS and 7.1% had PV. In univariable analysis disaggregated by gender, low income, poor education, perceived difficulty attending healthcare, and alcohol use were associated with VNS in men while low CD4 at diagnosis, multiple prior ART regimens and treatment interruptions were significant in both genders. Multiple prior ART regimens (adjusted Odds Ratio (aOR) 2.82, [95% confidence interval (CI) 1.59, 4.99], *p* < 0.01), treatment interruptions (aOR 4.51, [95% CI 2.13, 9.58], *p* < 0.01), excessive alcohol consumption (aOR 2.56, [95% CI 1.18, 5.54], *p* < 0.05) perceived difficulty attending healthcare (aOR 2.07, [ 95% CI 1.25, 3.42], *p* < 0.01) and low CD4 at diagnosis (aOR 2.34, 95% [CI 1.30, 4.20], *p* < 0.01) were independently associated with VNS on multivariable regression.

**Conclusions:**

We conclude that socio-economic and clinical factors influence viral suppression in our cohort and vary between men and women. Gender specific approaches are necessary to achieve the 90% suppression goal.

**Supplementary Information:**

The online version contains supplementary material available at 10.1186/s12981-021-00400-9.

## Background

Over the last two decades, huge strides have been made in the control of the HIV epidemic. This is widely attributed to the global scale-up in access to antiretroviral therapy (ART). In 2013, the Joint United Nations Programme of HIV/AIDS (UNAIDS) proposed the 90–90–90 goal as a benchmark to contribute to the end of the HIV epidemic. The goal was to diagnose 90% of people living with HIV (PLHIV), provide ART for 90% of those diagnosed and achieve viral suppression in 90% of those receiving ART by 2020 [1, 2]. There has been significant progress towards this goal, mainly driven by the efforts in south and east Africa. However, entire regions continue to experience setbacks in the fight against HIV. Such is the case of Latin America, where no countries have reached the 90–90–90 goal, HIV new infections increased by 7% in the past eight years and only 55% of all the diagnosed PLHIV are virally suppressed [2–4].

Viral suppression plays a crucial role in limiting onward transmission of HIV. As HIV prevalence increases, viral suppression must be achieved in order to prevent new infections [5]. Several studies have pointed out that viral suppression decreases the likelihood of HIV transmission in the at-risk and general population and improves overall life quality and life expectancy [6–8]. Individuals failing to achieve viral suppression may progress to persistent viremia, which is associated with double the risk of virologic failure and with higher rates of all-cause mortality [9–11].

The main concern in Latin America is that progress, while steady, is uneven between regions [2]. In Guatemala, the country with the highest number of PLHIV in Central America, 62% of PLHIV know their HIV status, 69% of all linked to care are taking ART and 80% taking ART are virally suppressed. In total, the percentage of all PLHIV in Guatemala with viral suppression is estimated at 34% [2, 3].

Several barriers to viral suppression have been identified and include but are not limited to stigma, difficulty in accessing healthcare because of location or financial status [12, 13], alcohol and substance misuse [2], education [14], pill burden [15], and ART side effects [16]. To our knowledge, a detailed analysis on factors that influence virologic outcomes in PLHIV has not been performed in Guatemala. We aimed to identify associated factors for virologic outcomes in the treatment of PLHIV in this Central American country.

## Methods

We used data from baseline interviews obtained as part of an ongoing prospective cohort study on comorbidities in PLHIV attending the Dr. Carlos Rodolfo Mejia Villatoro Infectious Diseases Clinic at Roosevelt Hospital in Guatemala City. The clinic is the largest HIV clinic in the country and provides free care for over 5000 PLHIV nationwide. PLHIV 18 years or older attending the HIV clinic are eligible for enrollment in the cohort. PLHIV were enrolled after providing verbal consent, and data was collected with in-depth interviews, clinical evaluation, and review of clinical files. The study was approved by the institutional review board of Roosevelt Hospital. The baseline interviews, containing questions on demographics, housing, medical conditions, and health risk factors, were conducted in Spanish and took place from July 2019 to February 2020. Study data were collected and managed using REDCap electronic data capture tools hosted at Washington University in Saint Louis [17].

### Study design

For the current analysis we included all PLHIV enrolled in the cohort that had been prescribed at least six consecutive months of ART since their initial diagnosis and had a recent viral load. Recent viral load was defined as a HIV viral load taken within three months prior or one month following the enrollment interview for participants with clinic visits every 3-months and viral load taken six months from enrollment at most for participants with 6-months clinic visits. Viral non-suppression (VNS) was defined as a viral load ≥ 200 copies/ml and persistent viremia (PV) was defined as two consecutive viral loads ≥ 200 copies/ml.

### Variable definitions

The continuous variables age, travel time and travel cost to HIV care, individual monthly income, years of education, number of prior ART regimens, CD4 count at diagnosis and days of treatment interruptions were recategorized into binary variables for analysis. Age 50 and below or over 50 years was used to compare younger and older adults, respectively, as defined by the Center for Disease Control and Prevention (CDC) [18]. Travel time to healthcare was defined as commutes within an hour and greater than an hour based on a prior study regarding accessibility in Guatemala [19]. Individual monthly income was evaluated by earning less than or equal to the “Canasta básica alimentaria” (CBA), or national food basket, which is the monthly cost to provide sufficient food for the average Guatemalan household. As of January 2020, the cost was $464 [20]. Education level was divided by PLHIV that achieved above primary education (first six years of formal education) from those with less or no formal education. Travel cost considered whether the round-trip cost to the clinic exceeded the median cost for our study population ($2.6). Multiple-daily dosing refers to more than once-daily dosing of ART. Treatment interruptions were defined as missing seven days or more of ART between changing regimens. Multiple prior ART regimens was defined as having three or more prior ART regimens.

Categorical variables were defined as follows. Excessive alcohol consumption was defined as binge drinking (four or more drinks in one occasion for women and five or more drinks for men) and heavy drinking (eight or more drinks in a week for women and 15 or more drinks in a week for men) within the past month, in accordance with the CDC definitions [21]. Access to basic utilities considered both running water and electricity in the participant’s residence. Indigenous participants were self-identified. Perceived difficulty attending healthcare was defined according to the participant’s yes/no response to the question “¿Le parece difícil asistir a la clínica?” Translation: “Do you find it difficult to visit the clinic?”. A follow up question gave participants the following reasons to choose from: lack of income, lack of transportation, nobody to take care of children, difficulty getting time off from work, price of transportation, or other reasons (free answer). Comorbidities were reported by participants and verified in the clinical file. Prior AIDS defining illnesses were assessed according to the CDC definitions [22].

### Statistical analysis

Statistical analyses were performed using SPSS (Statistical Package for the Social Sciences) software version 23. Baseline characteristics were analyzed with respect to frequency and percentage. Normality was evaluated for all numeric variables using Shapiro Wilk test with kurtosis and skewness. Baseline characteristics were compared with the nonparametric Pearson ${x}^{2}$ and Fisher’s exact test of independence for categorical variables and Mann–Whitney U test for continuous variables with interquartile range. Binary logistic regression was performed to explore the association between each variable and VNS and PV. Multivariable logistic regression models were fitted with variables at *p* ≤ 0.05 to evaluate independent factors associated with VNS and PV.

## Results

Of 929 participants enrolled in the cohort, 664 had a recent viral load and had completed at least six months of ART. The median age was 39 years [interquartile range (IQR) 32–48], 356 (53.6%) were male, 493 (74.2%) were heterosexual and 99 (14.9%) self-identified with indigenous ethnicity. Almost half of the participants (49.2%) had less than or equal to primary education and only 60 (9.0%) had a bachelor’s degree or higher. A total of 441 (66.4%) participants reported being employed. Out of these, 23.7% (158) were females and 42.6% (283) were males. Five hundred sixty had no income or earned less than the CBA with 211 (31.8%) having to borrow money to cover travel costs. The main transportation used was public buses (606, 91.3%). Travel time varied greatly, but 80 (12%) participants traveled four or more hours to reach healthcare.

Comorbidities were present in 408 (61.4%) and only 21 (3.2%) participants were co-infected with hepatitis B. Median CD4 count and viral load at diagnosis were 178 cells/mm^3^ (IQR 57–341) and 77,000 copies/ml (IQR 21,248–252,252) respectively. Current median CD4 count was 473 cells/mm3 (IQR 283–689). A total of 262 (39.5%) of our participants were in their first ART regimen, 402 (60.5%) had at least one prior ART regimen and 105 (15.8%) had multiple prior ART regimens. Other characteristics arranged by virologic status are presented in Table 1. VNS was present in 88 (13.3%) participants on recent HIV viral load testing. Differences between those with viral suppression and VNS are illustrated in Table 1. Of those who were not virally suppressed, 47 (7.1%) had PV.

**Table 1:** Characteristics of 664 virally suppressed and non-suppressed PLHIV on ART in Guatemala

Variable	Total (664)	Viral Suppression (576)	Viral Non-suppression (88)	p-value^a^
Age in years (IQR)	39 [32, 48]	40 [33, 48]	37.5 [34.5,44.5]	0.512
Male *n (%)*	356 (53.6%)	305 (53%)	51 (58%)	0.381
Sexual orientation *n (%)*	653 (98.3%)			0.016
MSM	112 (16.9%)	105 (18%)	7 (8%)	
Bisexual	48 (7.2%)	38 (7%)	10 (11%)	
Heterosexual	493 (74.2%)	425 (74%)	68 (77%)	
Non-indigenous ethnicity *n (%)*	565 (85.1%)	488 (85%)	77 (88%)	0.496
Years of education (IQR)	6 [3, 11]	7 [3, 11]	6 [4, 9]	0.164
Individual monthly income^b^ (IQR)	150 [0.375]	182 [0.389]	51 [0.318]	0.058
Working status	441 (66.4%)	391 (67.0%)	50 (56.8%)	0.052
Home owner *n (%)*	422 (63.6%)	362 (63%)	60 (68%)	0.333
Lack of access to basic utilities *n (%)*	80 (12.0%)	67 (12%)	13 (15%)	0.399
Smoking *n (%)*	77 (11.6%)	60 (10%)	17 (19%)	0.015
Excessive alcohol consumption *n (%)*	65 (9.8%)	51 (9%)	14 (16%)	0.038
Prior illicit drug use *n (%)*	70 (10.5%)	57 (10%)	13 (15%)	0.165
Travel time to healthcare in minutes (IQR)	90 [60, 150]	90 [45,150]	90 [60,180]	0.395
Borrows money for transport to care *n (%)*	211 (31.8%)	180 (31%)	31 (35%)	0.455
Round-trip travel cost^b^ (IQR)	2.5 [1.25, 7.5]	2.27 [1.3,6.49]	2.66 [1.04,8.04]	0.741
Perceived difficulty attending healthcare *n (%)*	258 (38.9%)	210 (37%)	48 (55%)	0.001
Participants with comorbidities *n (%)*	408 (61.4%)	363 (63%)	45 (51%)	
Dyslipidemia	298 (44.9%)	293 (44%)	5 (5%)	
Hypertension	59 (8.9%)	53 (9%)	6 (7%)	0.030
Diabetes	36 (5.4%)	32 (5.6%)	4 (4.5%)	
Past AIDS-defining illness *n (%)*	221 (33.3%)	178 (31%)	43 (49%)	0.001
Current tuberculosis diagnosis	3 (0.5%)	1 (0.2%)	2 (2.3%)	
CD4 count at diagnosis (IQR)	174 [57, 341]	178 [55,346]	58 [24.5,145.5]	0.000
Treatment interruptions ≥ seven days *n (%)*	42 (6.3%)	26 (5%)	16 (18%)	0.000
Multiple prior ART regimens *n (%)*	105 (15.8%)	74 (13%)	31 (35%)	0.000
Multiple-daily dosing *n (%)*	181 (27.2%)	142 (25%)	39 (44%)	0.001
Integrase inhibitor-based regimen *n (%)*	234 (35.2%)	196 (34%)	38 (43%)	0.094

MSM, men who have sex with men; ART, antiretroviral therapy; IQR, interquartile range

^a^Comparisons were calculated with Chi-squared test for categorical variables and Mann–Whitney U for continuous variables ^b^In dollars

On univariable analysis, excessive alcohol consumption, perceived difficulty attending healthcare, having no comorbidities, treatment interruptions, multiple prior ART regimens, multiple-daily dosing, low CD4 count at diagnosis, past AIDS-defining illness and sexuality were associated with VNS (*p* ≤ 0.05) and were included in the multivariable analysis (Table 2). In the multivariable analysis, treatment interruptions (aOR 4.51, [95% CI 2.13, 9.58], *p* < 0.01), multiple prior ART regimens (aOR 2.82, [95% CI 1.59, 4.99], *p* < 0.01), excessive alcohol consumption (aOR 2.56, [95% CI 1.18, 5.54], p = 0.017), low CD4 count at diagnosis (aOR 2.34, [95% CI 1.30, 4.20], p < 0.01), bisexual orientation (aOR 4.04, [95% CI 1.33,12.32], p = 0.014), and perceived difficulty attending healthcare (aOR 2.07, [95% CI 1.25, 3.42]*, p* < 0.01), were all associated with increased risk of VNS. Among those with perceived difficulty, the most commonly reported reasons for such difficulty were difficulty getting time off from work (72, 28.6%) and price of transportation (71, 27.5%) (Additional file 3: Table S3), but no association was found between specific reasons for perceived difficulty attending healthcare and VNS (Additional file 3: Table S3).

**Table 2:** Univariable and multivariable logistic regression of viral non-suppression in 647 PLHIV on ART in Guatemala

Variable	Univariable			Multivariable		
	aOR	95% CI	p-value	aOR	95% CI	p-value
Smoking	2.06	1.14–3.73	0.017	1.73	0.83–3.60	0.141
Excessive alcohol consumption	1.95	1.03–3.69	0.041	2.56	1.18–5.54	0.017
Perceived difficulty attending healthcare	2.09	1.33–3.29	0.001	2.07	1.25–3.42	0.005
No comorbidities	1.63	1.04–2.56	0.034	1.72	1.04–2.84	0.033
Past AIDS defining illness	2.14	1.36–3.36	0.001	1.23	0.72–2.11	0.450
Low CD4 count at diagnosis	2.55	1.54–4.22	0.000	2.34	1.30–4.20	0.004
Treatment interruption ≥ seven days	4.70	2.41–9.18	0.000	4.51	2.13–9.58	0.000
Multiple prior ART regimens	3.69	2.24–6.09	0.000	2.82	1.59–4.99	0.000
Multiple-daily dosing	2.43	1.53–3.86	0.000	1.59	0.92–2.74	0.095
Sexual orientation						
MSM	1.00		0.036	1.00		0.064
Bisexual	3.95	1.4–11.11	0.009	4.04	1.33–12.3	0.014
Heterosexual	2.40	1.07–5.38	0.033	1.61	0.66–3.91	0.298
Age 50 and below	1.11	0.62–1.98	0.723			
Male	1.23	0.78–1.93	0.381			
Not indigenous ethnicity	1.26	0.65–2.47	0.496			
Primary education or less	1.39	0.88–2.19	0.157			
Individual income ≤ CBA^a^	2.00	0.94–4.27	0.074			
Home owner	1.27	0.78–2.05	0.334			
Lack of access to basic utilities	1.32	0.69–2.50	0.400			
Prior illicit drug use	1.58	0.83–3.02	0.168			
Travel time to healthcare > 1 h	1.27	0.79–2.04	0.330			
Borrows money for transport to care	1.20	0.75–1.92	0.456			
Travel cost ≥ $2.6 roundtrip^b^	1.15	0.74–1.81	0.534			
Integrase inhibitor-based regimen	1.47	0.93–2.32	0.095			

*MSM* men who have sex with men, *ART* antiretroviral therapy, *aOR* adjusted odds ratio, *CI* confidence interval

Significant variables (*p* < 0.05) in univariable analysis were entered into multivariable analysis

^a^Monthly income ≤ *Canasta Básica Alimentaria*, the cost to feed an average Guatemalan household per month

^b^$2.6 was the median cost of transport reported in the cohort

Univariable analysis disaggregated by gender (Additional file 1: Table S1 and Additional file 2: Table S2), showed that men’s perceived difficulty attending healthcare was associated with VNS (aOR 3.05, [95% CI 1.66, 5.59], *p* < 0.01), but the same association was not seen in women (aOR 1.36, [95% CI 0.68,2.72], *p* = 0.371). Multiple-daily dosing (aOR 4.33, [95% CI 2.33, 8.04], *p* < 0.01), excessive alcohol consumption (aOR 2.19, [95% CI 1.02, 4.67] *p* = 0.042), income below or equal to the CBA (aOR 3.49, [95% CI 1.34,9.10], *p* = 0.010), and primary education or less (aOR 2.01, [95% CI 1.10,3.65], *p* = 0.022) were also significantly associated with VNS in men but not in women. Past AIDS defining illnesses, treatment interruptions, low CD4 count at diagnosis and having multiple prior ART regimens were still significantly associated with VNS in both genders in univariable analysis. In separate multivariable models however, only multiple prior ART regimens was significant in both men and women.

In the univariable analysis for PV as the outcome of interest, past AIDS defining illness, low CD4 count at diagnosis, treatment interruptions, multiple prior ART regimens, multiple-daily dosing, bisexual orientation, perceived difficulty attending healthcare, and having no comorbidities were significantly associated with PV. In the multivariable analysis, participants with multiple prior ART regimens (aOR 4.63, [95% CI 2.36, 9.09], *p* < 0.01), treatment interruptions (aOR 4.33, [95% CI 1.72,10.87], *p* < 0.01), and low CD4 count at diagnosis (aOR 2.36, [95% CI 1.07,5.22], *p* = 0.03) were associated with higher odds of PV, as shown in Table 3. These variables were also significantly associated with VNS.

**Table 3:** Univariable and multivariable logistic regression of persistent viremia in 647 PLHIV on ART in Guatemala

Variable	Univariable			Multivariable		
	aOR	95% CI	p-value	aOR	95% CI	p-value
Past AIDS-defining illness	2.62	1.43–4.79	0.002	1.46	0.73–2.91	0.281
Low CD4 count at diagnosis	3.21	1.56–6.60	0.001	2.36	1.07–5.22	0.033
Treatment interruption ≥ seven days	5.73	2.55–12.88	0.000	4.33	1.72–10.87	0.002
Multiple prior ART regimens	6.03	3.23–11.25	0.000	4.63	2.36–9.09	0.000
Multiple-daily dosing	2.24	1.22–4.11	0.009	1.39	0.70–2.76	0.335
Perceived difficulty attending healthcare	1.82	1.00–3.31	0.049	1.66	0.85–3.21	0.132
No comorbidities	1.91	1.05–3.48	0.033	1.92	0.99–3.70	0.051
Age 50 and below	1.09	0.51–2.33	0.809			
Male	1.54	0.83–2.85	0.163			
Not indigenous ethnicity	1.54	0.59–4.03	0.370			
Primary education or less	1.39	0.75–2.55	0.284			
Individual income ≤ CBA^a^	2.06	0.72–5.91	0.175			
Home owner	1.37	0.71–2.62	0.341			
No basic utilities access	1.32	0.57–3.09	0.509			
Prior illicit drug use	1.35	0.54–3.34	0.511			
Travel time to healthcare > 1 h	1.19	0.63–2.23	0.584			
Borrows money for transport to care	1.22	0.65–2.27	0.530			
Travel cost ≥ $2.6 roundtrip^b^	1.33	0.72–2.41	0.355			
Integrase inhibitor-based ART	1.22	0.66–2.27	0.514			
Sexual orientation						
MSM	1.00		0.123			
Bisexual	5.74	1.35–24.35	0.018			
Heterosexual	2.71	0.81–8.98	0.103			
Smoking	2.10	0.96–4.57	0.062			
Excessive alcohol consumption	1.51	0.61–3.75	0.371			

*MSM* men who have sex with men, *ART* antiretroviral therapy, *aOR* adjusted odds ratio, *CI* confidence interval

Significant variables (*p* < 0.05) in univariable analysis were entered into multivariable analysis

^a^monthly income ≤ *Canasta Básica Alimentaria*, the cost to feed an average Guatemalan household per month

^b^$2.6 was the median cost reported in the cohort

## Discussion

To our knowledge, a detailed analysis on factors that influence virologic outcomes in PLHIV has not been performed in Guatemala. Our study found factors associated with VNS and PV among PLHIV in Guatemala, complementing prior findings about the modifiable and unmodifiable factors associated with detectable viremia in PLHIV on ART [23–28]. Several studies have demonstrated that the risk for negative outcomes such as mortality, virologic failure and AIDS increases for PLHIV at low viremic levels, starting from 50 to 200 copies/ml [24, 29]. Therefore, 200 copies/ml was used in this study as a threshold to identify people with detectable viremia.

Perceived difficulty attending healthcare was one of the variables associated with VNS. Several socio-economic factors have been shown to have a negative impact on perception of HIV care such as quality of services, financial fairness, transportation convenience, perceived negative attitude, and stigma [30]. However, none of the reasons for perceived difficulty in our study was significantly associated with VNS, suggesting that each participant may have individualized difficulties that influence their viral load suppression.

We found that paradoxically, having no comorbidities increases the risk of VNS and PV. Several studies have found no association or a similar association to our study [31–33]. This may be explained by more frequent interactions with healthcare for people with comorbidities, due to their chronic conditions, where they may be encouraged to seek HIV care [34, 35].

In this study, indigenous ethnicity and age did not have an impact on VNS or PV. This differs from other studies where ethnic minorities [36, 37] and younger populations [10, 27, 37, 38] are more likely to experience worse outcomes in HIV care. Our findings may have been influenced by the low frequency of older PLHIV and those who self-identify as indigenous in our cohort, and further studies are needed in these populations.

Gender was also not associated with VNS in the overall population, in keeping with previous research on gender and detectable viremia [39, 40]. However, analysis disaggregated by gender showed important differences (Additional file 1: Table S1 and Additional file 2: Table S2). Lower individual income, low educational attainment, engaging in risk behaviors, bisexual orientation and perceived difficulty attending healthcare exclusively had an impact on viremia in men. Individual income may be less relevant in women due to differences in workforce participation in the cohort (23.7% in women vs 42.6% in men) [41]. Other studies have examined the interplay between gender, HIV and risk behaviors and have observed that the prevalence of heavy alcohol consumption is higher in men with HIV than in women [42], at-risk drinking in men is predictive of virologic failure [43], and MSM who engage in higher risk drinking are more likely to engage in HIV risk behaviors [44]. We emphasize these gender differences due to our concern that men are getting left behind in the fight against the HIV epidemic [45] which calls to revise policies regarding men’s sexual health services.

Unlike VNS, we found no social or economic variables associated with PV. However, the same variables related to HIV background were significantly associated with both VNS and PV. Having multiple prior ART regimens was independently associated with both outcomes with the highest odds ratio of all relevant variables. Similar findings are reported in South Africa [46], Uganda [47], and the United States [48] where having multiple prior ART regimens or being on 2nd line therapy was significantly associated with PV. We agree with other hypotheses that multiple ART changes are more likely associated with poor adherence, acquired drug resistance mutations, and long-term drug toxicities that influence treatment efficacy and viral suppression [46–48]. Not all studies support this link between the viral suppression and ART history [49], but this finding should encourage further research on reasons for multiple ART changes and HIV outcomes in Guatemala, especially since the HIV treatment guidelines were updated in December 2019 to include integrase inhibitors as first line of therapy [50, 51].

Low CD4 count at diagnosis was another variable significantly associated with VNS and PV, similar to what was found in several cohort studies [23, 52–54]. Progressing to advanced disease before treatment may put patients at higher risk of persistent viremia once treatment is started as a consequence of increased viral reservoir size, but further research is needed to understand this association.

We also demonstrate that treatment interruptions are associated with VNS as well as PV. This result is similar to what is reported from multiple studies where missing any amount of days of ART is significantly associated with PV [25, 27, 38, 47]. Other studies have related treatment interruptions and poor adherence to poor education, HIV stigma, low socioeconomic status, risk behaviors, treatment fatigue, and young age [55, 56]. These treatment interruptions may occur due to lack of HIV care facilities or treatment support programs in Guatemala, in addition to individual challenges in attending care. This could be a key area of intervention to impact both the percentage of PLHIV taking ART and the proportion of those that are virally suppressed.

There are important limitations in our study. Although all participants were selected randomly, a small number of people declined participation mainly due to insufficient time to conduct an interview. Those that were temporarily lost to follow up in HIV care, hospitalized in-patient, or attending prenatal care were not captured in this cohort. Adolescents were not considered due to health policies regarding age and sexual health consent. Transgender population and sex workers are underrepresented in our cohort due to the lack of public outreach and gender specific programs [2]. We were unable to include HIV subtypes and clades for our participants because this data is not available as standard of care. However, one cohort study previously published from our clinic showed that 98.9% of sequences performed belong to B_Pandemic_ clade [57]. Treatment interruptions were only captured in participants lost to follow up for more than three months or with ART that differed in at least one drug or differed in doses, but not in those that did not change regimen or dosing. During treatment interruptions some participants may share treatment with a partner or split pills. For prior ART switches, we were able to accurately capture reasons for treatment switch due to lack of prior information for some of the files. Household income, which was not available in this data, may be a better measurement of economic status than individual income.

## Conclusions

This is the first study, to our knowledge, demonstrating potential social and clinical influences on VNS and PV in PLHIV in Guatemala. In our analysis, we found that social factors such as excessive alcohol consumption, perceived difficulty attending healthcare, and bisexual orientation were significantly associated with VNS in PLHIV in Guatemala. In this cohort low CD4 count at diagnosis, treatment interruptions, multiple prior ART regimens, and multiple-daily dosing were significantly associated with both VNS and PV. This highlights potential areas of improvement to advance towards achieving the goal of 90% suppression. Simplifying regimens when possible, addressing personal barriers, improving access to ART outside of the clinic, and concentrating efforts on groups at greater risk are key interventions to continue improving viral suppression. The strong link between perceived difficulty attending healthcare and VNS warrants further exploration, as PLHIV often need to overcome barriers to optimal care.

## Supplementary Information

**Additional file 1: Table S1.** Univariable and multivariable logistic regression of viral non-suppression in 308 women^†^ living with HIV on ART in Guatemala.

**Additional file 2: Table S2.** Univariable and multivariable logistic regression of viral non-suppression in 356 men living with HIV on ART in Guatemala.

**Additional file 3: Table S3.** Reasons for perceived difficulty attending HIV care in 258 PLHIV on ART in Guatemala.

## Data Availability

The datasets generated and/or analyzed during the current study are available in the figshare.com repository: 10.6084/M9.FIGSHARE.13218806[58].

## Abbreviations

AIDS: Acquired immunodeficiency syndrome
aOR: Adjusted odds ratio
ART: Antiretroviral therapy
CBA: *Canasta básica alimentaria* (In English, national food basket)
CDC: Center for Disease Control
CI: Confidence interval
PLHIV: People living with HIV
HIV: Human immunodeficiency virus
IQR: Interquartile range
MSM: Men who have sex with men
PV: Persistent viremia
UNAIDS: Joint United Programme on HIV/AIDS
VNS: Viral non-suppression

## References

[1] UNAIDS (2014). 90–90–90: an ambitious treatment target to help end the AIDS epidemic.

[2] UNAIDS (2019). UNAIDS Data 2019.

[3] UNAIDS (2020). Advancing towards 2020: progress in Latin America and the Caribbean.

[4] Costa JM, Torres TS, Coelho LE, Luz PM (2018). Adherence to antiretroviral therapy for HIV/AIDS in Latin America and the Caribbean: systematic review and meta-analysis. J Int AIDS Soc.

[5] CDC. HIV Treatment as prevention: center for disease control and prevention; 2020. [Updated 2020–04–06T05:07:42Z]. https://www.cdc.gov/hiv/risk/art/index.html. Accessed 12 Aug 2020.

[6] Albert J, Berglund T, Gisslén M (2014). Risk of HIV transmission from patients on antiretroviral therapy: a position statement from the Public Health Agency of Sweden and the Swedish Reference Group for Antiviral Therapy. Scand J Infect Dis.

[7] Rodger AJ, Cambiano V, Bruun T (2016). Sexual activity without condoms and risk of HIV transmission in serodifferent couples when the HIV-positive partner is using suppressive antiretroviral therapy. JAMA.

[8] Bavinton BR, Pinto AN, Phanuphak N (2018). Viral suppression and HIV transmission in serodiscordant male couples: an international, prospective, observational, cohort study. Lancet HIV.

[9] Elvstam O, Medstrand P, Yilmaz A, Isberg PE, Gisslén M, Björkman P (2017). Virological failure and all-cause mortality in HIV-positive adults with low-level viremia during antiretroviral treatment. PLoS ONE.

[10] Carriquiry G, Giganti MJ, Castilho JL (2018). Virologic failure and mortality in older ART initiators in a multisite Latin American and Caribbean Cohort. J Int AIDS Soc.

[11] Bezabhe WM, Chalmers L, Bereznicki LR, Peterson GM (2016). Adherence to antiretroviral therapy and virologic failure: a meta-analysis. Medicine.

[12] Ridgway JP, Friedman EE, Choe J, Nguyen CT, Schuble T, Pettit NN (2020). Impact of mail order pharmacy use and travel time to pharmacy on viral suppression among people living with HIV. AIDS Care.

[13] Joy R, Druyts EF, Brandson EK (2008). Impact of neighborhood-level socioeconomic status on HIV disease progression in a universal health care setting. JAIDS J Acquir Immune Defic Syndr.

[14] Diress G, Dagne S, Alemnew B, Adane S, Addisu A (2020). Viral Load Suppression after enhanced adherence counseling and its predictors among high viral load HIV seropositive people in North Wollo Zone Public Hospitals, Northeast Ethiopia, 2019: retrospective cohort study. AIDS Res Treat.

[15] Scott Sutton S, Magagnoli J, Hardin JW (2016). Impact of pill burden on adherence, risk of hospitalization, and viral suppression in patients with HIV infection and AIDS receiving antiretroviral therapy. Pharmacother J Hum Pharmacol Drug Ther.

[16] Ammassari A, Murri R, Pezzotti P (2001). Self-reported symptoms and medication side effects influence adherence to highly active antiretroviral therapy in persons with HIV infection. J Acquir Immune Defic Syndr.

[17] Harris PA, Taylor R, Thielke R, Payne J, Gonzalez N, Conde JG (2009). Research electronic data capture (REDCap)—a metadata-driven methodology and workflow process for providing translational research informatics support. J Biomed Inform.

[18] CDC. HIV among people aged 50 and over: CDC; 2020. [Updated 2020–06–04T05:30:43Z]. https://www.cdc.gov/hiv/group/age/olderamericans/index.html. Accessed 15 Aug 2020.

[19] Annis S (1981). Physical access and utilization of health services in rural Guatemala. Soc Sci Med Part D Med Geogr.

[20] INE (2020). Canasta Básica Alimentaria (CBA) y Canasta Ampliada (CA) Enero 2020.

[21] CDC. Preventing excessive alcohol use [Updated 2020–04–08T02:04:20Z]. https://www.cdc.gov/alcohol/fact-sheets/prevention.htm. Accessed 03 Sept 2020.

[22] Selik R, Mokotoff E, Branson B, Owen M, Whitmore S, Hall H (2014). Revised Surveillance Case Definition for HIV Infection - United States, 2014. MMWR Recomm Rep Morbidity Mortality Weekly Rep Recomm Rep Centers Dis Control.

[23] Mpondo BC, Kilonzo SB, Meda JR, Gunda DW (2015). Prevalence and predictors of immunological failure among HIV-infected adults on HAART in Northwestern Tanzania: a cross sectional study. Tanzania Med J.

[24] Esber A, Polyak C, Kiweewa F (2018). Persistent low-level viremia predicts subsequent virologic failure: is it time to change the third 90?. Clin Infect Dis.

[25] Leierer G, Grabmeier-Pfistershammer K, Steuer A, Geit M, Sarcletti M, Haas B (2015). Factors associated with low-level viraemia and virological failure: results from the Austrian HIV cohort study. PLoS ONE.

[26] Maldonado-Martínez G, Hunter-Mellado RF, Fernández-Santos D, Ríos-Olivares E (2015). Persistent HIV viremia: description of a cohort of HIV infected individuals with ART failure in Puerto Rico. Int J Environ Res Public Health.

[27] Atuhaire P, Hanley S, Yende-Zuma N (2019). Factors associated with unsuppressed viremia in women living with HIV on lifelong ART in the multi-country US-PEPFAR PROMOTE study: a cross-sectional analysis. PLoS ONE.

[28] Sinai IB, C. Cantelmo, R. Mbuya-Brown, Y. Panjshiri, and M. Balampama. Adolescent HIV in Tanzania: factors affecting viral load suppression and the transition to adult care. Washington, DC: Palladium, Health policy plus; 2020. http://www.healthpolicyplus.com/ns/pubs/13334-13611_TanzaniaPediatricHIVReport.pdf. Accessed 10 Dec 2020.

[29] Laprise C, de Pokomandy A, Baril J-G, Dufresne S, Trottier H (2013). Virologic failure following persistent low-level viremia in a cohort of HIV-positive patients: results from 12 years of observation. Clin Infect Dis.

[30] Yakob B, Ncama BP (2016). Correlates of strengthening lessons from HIV/AIDS treatment and care services in Ethiopia perceived access and implications for health system. PLoS ONE.

[31] Ahn MY, Jiamsakul A, Khusuwan S (2019). The influence of age-associated comorbidities on responses to combination antiretroviral therapy in older people living with HIV. J Int AIDS Soc.

[32] Jimenez H, Stevens T, Suh J (2018). 581. Do comorbidities and polypharmacy lead to virologic failure in all populations living with HIV?. Open Forum Infect Dis.

[33] Rodriguez-Penney AT, Iudicello JE, Riggs PK (2013). Co-morbidities in persons infected with HIV: increased burden with older age and negative effects on health-related quality of life. AIDS Patient Care STDS.

[34] Browne J, Edwards DA, Rhodes KM, Brimicombe DJ, Payne RA (2017). Association of comorbidity and health service usage among patients with dementia in the UK: a population-based study. BMJ Open.

[35] van Oostrom S, Picavet S, de Bruin S (2014). Multimorbidity of chronic diseases and health care utilization in general practice. BMC Fam Pract.

[36] Kassaye SG, Wang C, Ocampo JMF, et al. Viremia trajectories of HIV in HIV-positive women in the United States, 1994–2017. (2574–3805 (Electronic)). 10.1001/jamanetworkopen.2019.3822 PMC6537820 31099865

[37] Crepaz N, Tang T, Marks G, Mugavero MJ, Espinoza L, Hall HI (2016). Durable viral suppression and transmission risk potential among persons with diagnosed HIV infection: United States, 2012–2013. Clin Infect Dis.

[38] Cherutich P, Kim AA, Kellogg TA (2016). Detectable HIV viral load in Kenya: data from a population-based survey. PLoS ONE.

[39] Rangarajan S, Donn JC, le Giang T (2016). Factors associated with HIV viral load suppression on antiretroviral therapy in Vietnam. J Virus Erad.

[40] Kroidl A, Burger T, Urio A (2020). High turnaround times and low viral resuppression rates after reinforced adherence counselling following a confirmed virological failure diagnostic algorithm in HIV-infected patients on first-line antiretroviral therapy from Tanzania. Trop Med Int Health.

[41] Guerra Morales N, Gomez Gomez F, Mancia Chua C (2017). ENEI 1–2019 Encuesta Nacional de Empleo e Ingresos.

[42] Ikeda ML, Barcellos NT, Alencastro PR (2016). Alcohol drinking pattern: a comparison between HIV-infected patients and individuals from the general population. PLoS ONE.

[43] Deiss RG, Mesner O, Agan BK (2016). Characterizing the association between alcohol and hiv virologic failure in a military cohort on antiretroviral therapy. Alcohol Clin Exp Res.

[44] Washington TA, Patel SN, Meyer-Adams N (2017). Drinking patterns and HIV risk behaviors among black and latino men who have sex within Los Angeles County. Am J Mens Health.

[45] Auld AF, Shiraishi RW, Mbofana FR, Couto A, Fetogang EB, El-Halabi S (2015). Lower levels of antiretroviral therapy enrollment among men with HIV compared with women—12 countries, 2002–2013.

[46] Osler M, Hilderbrand K, Goemaere E (2018). The continuing burden of advanced HIV disease over 10 years of increasing antiretroviral therapy coverage in South Africa. Clin Infect Dis.

[47] Kiweewa F, Esber A, Musingye E (2019). HIV virologic failure and its predictors among HIV-infected adults on antiretroviral therapy in the African Cohort Study. PLoS ONE.

[48] Fleming J, Mathews WC, Rutstein RM (2019). Low-level viremia and virologic failure in persons with HIV infection treated with antiretroviral therapy. AIDS.

[49] Buchacz K, Wiegand R, Armon C (2015). Long-term immunologic and virologic responses on raltegravir-containing regimens among ART-experienced participants in the HIV Outpatient Study. HIV Clin Trials.

[50] Programa Nacional de Prevención y Control de ITS, VIH y SIDA (2013). Guía de tratamiento antirretroviral y de infecciones oportunistas en Guatemala 2013.

[51] Programa Nacional de Prevención y Control de ITS, VIH y SIDA. Guía de uso de los antirretrovirales y en personas con VIH y su aplicación profiláctica 2019. Ciudad de Guatemala: Ministerio de Salud Pública y Asistencia Social; 2019. p. 73. https://www.mspas.gob.gt/component/jdownloads/send/410-guias/2765-guia-de-uso-de-los-antirretrovirales-en-personas-con-vih-y-su-aplicacio-n-profila-ctica.html.

[52] Joya C, Won SH, Schofield C (2019). Persistent Low-level viremia while on antiretroviral therapy is an independent risk factor for virologic failure. Clin Infect Dis.

[53] Hermans LE, Carmona S, Nijhuis M (2020). Virological suppression and clinical management in response to viremia in South African HIV treatment program: a multicenter cohort study. PLoS Med.

[54] Zhang T, Ding H, An M (2020). Factors associated with high-risk low-level viremia leading to virologic failure: 16-year retrospective study of a Chinese antiretroviral therapy cohort. BMC Infect Dis.

[55] Bukenya D, Mayanja BN, Nakamanya S, Muhumuza R, Seeley J (2019). What causes non-adherence among some individuals on long term antiretroviral therapy? Experiences of individuals with poor viral suppression in Uganda. AIDS Res Ther.

[56] Kim J, Lee E, Park B-J, Bang JH, Lee JY (2018). Adherence to antiretroviral therapy and factors affecting low medication adherence among incident HIV-infected individuals during 2009–2016: a nationwide study. Sci Rep.

[57] Mendoza Y, García-Morales C, Bello G, Garrido-Rodríguez D, Tapia-Trejo D, Pascale J (2018). Evolutionary history and spatiotemporal dynamics of the HIV-1 subtype B epidemic in Guatemala. PLoS ONE.

[58] Ortiz D, Roberts-Sano O, O’Halloran J (2020). Figshare.

