# Supplementary material for: Factors associated with viremia in people living with HIV on antiretroviral therapy in Guatemala
Source: AIDS Res Ther. 2021 Oct 27;18:79. doi: 10.1186/s12981-021-00400-9 (PMC8554948; doi:10.1186/s12981-021-00400-9)
Supplement: Supplementary file 1 — Additional file 1: Table S1. Univariable and multivariable logistic regression of viral non-suppression in 308 women† living with HIV on ART in Guatemala. [file 12981_2021_400_MOESM1_ESM.docx]

| **Table S1. Univariable and multivariable logistic regression of viral non-suppression in 298 women**^†^ **living with HIV on ART in Guatemala.** | | | | | | |
| --- | --- | --- | --- | --- | --- | --- |
|  | **Univariable** | | | **Multivariable** | | |
| **Variable** | **aOR** | **95% CI** | **p-value** | **aOR** | **95% CI** | **p-value** |
| Past AIDS defining illness | 2.30 | 1.14-4.617 | 0.019 | 1.60 | 0.76-3.38 | 0.213 |
| Low CD4 cell count at diagnosis | 2.20 | 1.04-4.65 | 0.038 | 1.68 | 0.76-3.69 | 0.194 |
| Treatment interruption ≥ seven days | 6.37 | 2.50-16.25 | 0.000 | 5.33 | 1.99-14.23 | 0.001 |
| Multiple prior ART regimens | 2.50 | 1.20-5.19 | 0.014 | 2.12 | 0.98-4.58 | 0.055 |
| Current smoker | 2.56 | 0.66-9.95 | 0.172 |  |  |  |
| Excessive alcohol consumption | 1.31 | 0.36-4.73 | 0.672 |  |  |  |
| Perceived difficulty attending healthcare | 1.36 | 0.68-2.72 | 0.371 |  |  |  |
| No comorbidities | 1.45 | 0.72-2.92 | 0.289 |  |  |  |
| Multiple-daily dosing | 1.22 | 0.59-2.52 | 0.579 |  |  |  |
| Age 50 and below | 1.46 | 0.58-3.68 | 0.412 |  |  |  |
| Not-indigenous ethnicity | 3.39 | 0.78-14.62 | 0.101 |  |  |  |
| Primary education or less | 0.94 | 0.46-1.92 | 0.877 |  |  |  |
| Individual income ≤ CBA ‡ | 2.00 | 0.94-4.27 | 0.074 |  |  |  |
| Home owner | 1.32 | 0.64-2.70 | 0.447 |  |  |  |
| Lack of access to basic utilities | 0.90 | 0.33-2.45 | 0.840 |  |  |  |
| Prior illicit drug use | 1.37 | 0.17-11.09 | 0.762 |  |  |  |
| Travel time to healthcare > 1hr | 1.08 | 0.51-2.30 | 0.831 |  |  |  |
| Borrows money for transport to care | 1.11 | 0.55-2.21 | 0.763 |  |  |  |
| Travel cost ≥ $2.6 roundtrip § | 1.22 | 0.61-2-43 | 0.571 |  |  |  |
| Integrase inhibitor-based regimen | 1.93 | 0.96-3.87 | 0.061 |  |  |  |
| ART, antiretroviral therapy; aOR, adjusted odds ratio; CI confidence interval  Significant variables (*p* < 0.05) in univariable analysis were entered into multivariable analysis. ^†^Cisgender women: 302, transgender women: 6, ‡monthly income ≤ *Canasta Básica Alimentaria*, the cost to feed an average Guatemalan household per month. ‡ $2.6 was the median cost of transport reported in the cohort. | | | | | | |
