# Supplementary material for: Factors associated with viremia in people living with HIV on antiretroviral therapy in Guatemala
Source: AIDS Res Ther. 2021 Oct 27;18:79. doi: 10.1186/s12981-021-00400-9 (PMC8554948; doi:10.1186/s12981-021-00400-9)
Supplement: Supplementary file 2 — Additional file 2: Table S2. Univariable and multivariable logistic regression of viral non-suppression in 356 men living with HIV on ART in Guatemala. [file 12981_2021_400_MOESM2_ESM.docx]

| **Table S2. Univariable and multivariable logistic regression of viral non-suppression in 339 men living with HIV on ART in Guatemala.** | | | | | | |
| --- | --- | --- | --- | --- | --- | --- |
|  | **Univariable** | | | **Multivariable** | | |
| **Variable** | **aOR** | **95% CI** | **p-value** | **aOR** | **95% CI** | **p-value** |
| Perceived difficulty attending healthcare | 3.05 | 1.66-5.59 | 0.000 | 2.56 | 1.31-4.99 | 0.006 |
| Low CD4 cell count at diagnosis | 2.84 | 1.43-5.64 | 0.003 | 2.10 | 0.98-4.49 | 0.055 |
| Multiple prior ART regimens | 6.76 | 3.25-14.05 | 0.000 | 4.81 | 2.12-10.90 | 0.000 |
| Multiple-daily dosing | 4.33 | 2.33-8.04 | 0.000 | 2.69 | 1.33-5.44 | 0.006 |
| Sexual orientation | 2.66 | 1.31-5.39 | 0.006 | 1.16 | 0.51-2.6 | 0.710 |
| Current smoker | 1.88 | 0.95-3.73 | 0.070 |  |  |  |
| Excessive alcohol consumption | 2.19 | 1.02-4.67 | 0.042 |  |  |  |
| No comorbidities | 1.73 | 0.95-3.14 | 0.070 |  |  |  |
| Past AIDS defining illness | 2.00 | 1.09-3.642 | 0.023 |  |  |  |
| Treatment interruption ≥ seven days | 3.57 | 1.32-9.44 | 0.010 |  |  |  |
| Age 50 and below | 0.88 | 0.41-1.87 | 0.743 |  |  |  |
| Not Indigenous ethnicity | 0.78 | 0.35-1.72 | 0.551 |  |  |  |
| Primary education or less | 2.01 | 1.10-3.65 | 0.022 |  |  |  |
| Individual income ≤ CBA † | 3.49 | 1.34-9.10 | 0.010 |  |  |  |
| Home owner | 1.18 | 0.62-2.27 | 0.602 |  |  |  |
| Lack of access to basic utilities | 1.91 | 0.81-4.49 | 0.135 |  |  |  |
| Prior illicit drug use | 1.68 | 0.82-3.46 | 0.152 |  |  |  |
| Travel time to healthcare > 1hr | 1.66 | 0.89-3.07 | 0.107 |  |  |  |
| Borrows money for transport to care | 1.64 | 0.80-3.37 | 0.172 |  |  |  |
| Travel cost ≥ $2.6 roundtrip‡ | 1.53 | 0.84-2.78 | 0.155 |  |  |  |
| Integrase inhibitor-based regimen | 1.19 | 0.64-2.19 | 0.569 |  |  |  |
| MSM, men who have sex with men; ART, antiretroviral therapy; aOR, adjusted odds ratio; CI confidence interval. For men, *p-value* for significant variables was set at < 0.01 to avoid overfitting the multivariable model. † monthly income ≤ *Canasta Básica Alimentaria*, the cost to feed an average Guatemalan household per month. ‡ $2.6 was the average cost of transport roundtrip. | | | | | | |
