# Supplementary material for: Factors associated with viremia in people living with HIV on antiretroviral therapy in Guatemala
Source: AIDS Res Ther. 2021 Oct 27;18:79. doi: 10.1186/s12981-021-00400-9 (PMC8554948; doi:10.1186/s12981-021-00400-9)
Supplement: Supplementary file 3 — Additional file 3: Table S3. Reasons for perceived difficulty attending HIV care in 258 PLHIV on ART in Guatemala. [file 12981_2021_400_MOESM3_ESM.docx]

**Table S3. Reasons for perceived difficulty attending HIV care in 258 PLHIV in Guatemala.**

| **Variable** | **Viral Suppression (210)** | **Viral Non-suppression (48)** | **p-value** |
| --- | --- | --- | --- |
| Lack of income | 24 (11.4%) | 13 (27.1%) | 0.113 |
| Lack of transportation | 15 (7.1%) | 2 (4.2%) | 0.199 |
| Nobody to take care of children | 6 (2.9%) | 1 (2.1%) | 0.636 |
| Difficulty getting time off from work | 60 (28.6%) | 12 (25.0%) | 0.729 |
| Price of transportation | 57 (27.1%) | 14 (29.2%) | 0.451 |
| Other reasons | 48 (22.9%) | 6 (12.5%) | 0.083 |

Other reasons were related to traffic, stigma, scheduling difficulties, long distance, dangerous location of the Hospital, complicated requirements to receive care at the clinic and having other appointments at other clinics.
